# Pulmonary lesions: correlative study of dynamic triple-phase enhanced CT perfusion imaging with tumor angiogenesis and vascular endothelial growth factor expression

**DOI:** 10.1186/s12880-021-00692-3

**Published:** 2021-10-30

**Authors:** Mingyue Zou, Zhenhua Zhao, Bingqian Zhang, Haijia Mao, Yanan Huang, Cheng Wang

**Affiliations:** 1 Department of Radiology, Shaoxing People’s Hospital (Shaoxing Hospital, Zhejiang University School of Medicine), Shaoxing, 312000 China; 2 Department of Pathology, Shaoxing People’s Hospital (Shaoxing Hospital, Zhejiang University School of Medicine), Shaoxing, 312000 China

**Keywords:** Lung cancer, Tomography, Perfusion imaging, Angiogenesis

## Abstract

**Background:**

To investigate value of the quantitative perfusion parameters of dynamic triple-phase enhanced CT in differential diagnosis of pulmonary lesions, and explore the correlation between perfusion parameters of lung cancer with microvessel density (MVD) and vascular endothelial growth factor (VEGF).

**Methods:**

73 consecutive patients with lung lesions who successfully underwent pre-operative CT perfusion examination with dynamic triple-phase enhanced CT and received a final diagnosis by postoperative pathology or a clinical follow-up. The cases were divided into malignant and benign groups according to the pathological results. CT perfusion parameters, such as Median, Mean, Standard deviation (Std), Q10, Q25, Q50, Q75, Q90 of pulmonary artery perfusion (PAP), bronchial artery perfusion (BAP), perfusion index (PI) and arterial enhancement fraction (AEF) were obtained by performing computed tomography perfusion imaging (CTPI). Computed tomography perfusion (CTP) parameters were compared between malignant and benign lesions. The receiver operating characteristic (ROC) curve was used to assess the diagnostic efficiency of CTP parameters in diagnosing malignant lesions. The correlations between CTP parameters with MVD and VEGF were analysed in 36 lung cancer patients who had extra sections be used for immunohistochemistry staining of CD34 and VEGF.

**Results:**

BAP (Mean, Std, Q90) and PI Std of benign lesions were higher than malignant lesions (*p* < 0.05), and PAP (Q10, Q25), PI (Median, Mean, Q10, Q25, Q50) of malignant lesions were higher than the benign (*p* < 0.05). The area under the ROC curve of PI Mean, PI Q10 and PI Std was 0.722 (95% CI = [0.595–0.845]), 0.728 (95% CI = [0.612–0.844]) and 0.717 (95% CI = [0.598–0.835]) respectively. Partial perfusion parameters of BAP and AEF Q10 were positively correlated with MVD (*p* value range is < 0.001–0.037, ρ value range is 0.483–0.683), and partial perfusion parameters of PI were negatively correlated with MVD (*p* value range is 0.001–0.041,ρvalue range is − 0.523–− 0.343). Partial perfusion parameters of BAP and AEF Q10 were positively correlated with VEGF (*p* value range is 0.001–0.016, ρvalue range is 0.398–0.570), meanwhile some perfusion parameters of PAP and PI were negatively correlated with VEGF (*p* value range is 0.001–0.040, ρ value range is − 0.657–0.343).

**Conclusions:**

Quantitative parameters of dynamic triple-phase enhanced CT can provide diagnostic basis for the differentiation of lung lesions, and there were connection with tumor angiogenesis and vascular endothelial growth factor expression.

## Background

Lung cancer is one of the common malignant tumors that seriously threatens us, and it is also the leading and increasing cause of cancer-related among men and women [1, 2]. The five-year survival rate of patients with early lung cancer can reach up to 54–73%, while patients with advanced lung cancer have a poor prognosis and a five-year survival rate only 2%[3, 4]. Therefore, early diagnosis of cancer patients is the basis for further rational treatment, which is directly related to the prognosis of patients.

Tumor microvessel formation and growth are affected by various factors, vascular endothelial growth factor (VEGF) is one of the most important regulatory factors. This is the strongest known growth factor that directly affecting vascular endothelial, which is a key mediator of tumor angiogenesis, promoting cancer cell proliferation and tumor microvascular grow, consisting the microcirculation state of lung cancer [5, 6]. Microvessel density (MVD) is recognized as the "gold standard" for judging angiogenesis, being used as an indicator to quantify the degree of angiogenesis[7]. Microvascular status in tumor tissues is associated with the expression of VEGF, the status of neovascularization and cell proliferation can be evaluated by detecting tumor microvascular parameters in lung cancer in order to infer the expression of tumor cytokines that causing tumor cell and vascular grow[8, 9]. The research of tumor angiogenesis and angiogenesis inhibitors is the focus and research hotspot of tumor prevention and treatment. So far, a variety of anti-vascular drugs have been developed and approved for clinical application, mostly for the treatment of advanced non-small cell lung cancer and metastatic tumors[10]. These drugs can normalize the abnormal blood vessels of the tumor and inhibit the formation of new blood vessels, thereby achieving the purpose of anti-tumor treatment[11]. Detection of MVD and VEGF expression require tissue-dependent testing. The technique is invasive and making it impractical for monitoring treatment, in addition it is also limited by random sampling errors and interobserver variability[12, 13]. Traditional imaging is difficult to show the microvessel in tumor tissue early, conventional unenhanced CT scan and dynamic enhancement only provide morphological and blood supply characteristics for the diagnosis of lung nodules, morphological indicators have some limitations in the differentiation of pulmonary lesions[14, 15]. CT perfusion imaging (CTPI) can reflect the tumor angiogenesis non-invasively, which also has important application value in quantitative and qualitative research of tumor [16, 17], widely used in the diagnosis of heart, brain and other organs[18, 19]. However, the application of dual-inputs perfusion with double blood supply organ like lung is still in the stage of continuous study. Previous perfusion CT has been limited because of its higher X-ray dose, but dynamic triple-phase enhanced CT only needs an additional one-stage scan. Our study is to evaluate the feasibility and reliability of perfusion quantitative parameters based on dynamic triple-phase enhanced CT in the diagnosis of lung lesions, to explore whether the parameters are related to the expression of VEGF and MVD, in order to seek a both safe and non-invasive method to evaluate the expression of VEGF and MVD in lung cancer.

## Material and method

### Patients

A total of 73 consecutive patients (51 male and 22 female, mean age = 65 ± 11 years) with known lung lesions confirmed in previous exams were recruited between November 2018 to July 2019. All patients were enrolled according to the following criteria: (a) Patients were suspected to diagnose as malignant lesions; (b) Clinical consideration inflammatory lesions or tuberculosis, the lesion disappeared or significantly absorbed in follow-up CT within 1 month; (c) No treatment before CT examination; (d) Lung lesions were mixed or subsolid (diameter ≥ 2 cm). Exclusion criteria:(a) Patients with contraindications for surgery or puncture; (b) Poor image quality; (c) the interval between operation or biopsy and CT examination time was more than 1 month. Because some of the patients who were punctured had no extra sections for experimental immunohistochemistry, there were 36 cases of lung cancer used for immunohistochemistry. The protein expression molecular imaging data were statistically analyzed, including 27 cases of male and 9 cases of female,49–85 (68 ± 9) years old. This study was approved by the ethics committee of Shaoxing people’s hospital.

### CT perfusion imaging protocol

All patients received breath training before performing the CT scanning with a 64–detector row scanner (Brilliance CT 64 Slice of Philips Corporation). The scanning protocol was a rotation time of 0.5 s, beam collimation of 64 X 0.625 mm, reconstruction section thickness was 2 mm and interval of 5.0 mm, pitch factor of 1.2 mm, default field of view (FOV) of 35 × 35 cm, tube voltage was 120 kV, tube current was 80mAs. Pulmonary artery phase scan was performed 15 s after injection of contrast material, aortic phase scan was performed 30 s, then delayed scan was performed 1 min later. In total, 80 ml contrast material (Ioversol, Jiangsu Hengrui Medicine Co) at a rate of 5.0 mL/s were injected with a 20-gauge catheter inserted into an antecubital vein by using a power injector.

### Image processing and analysis

**Fig. 1:**
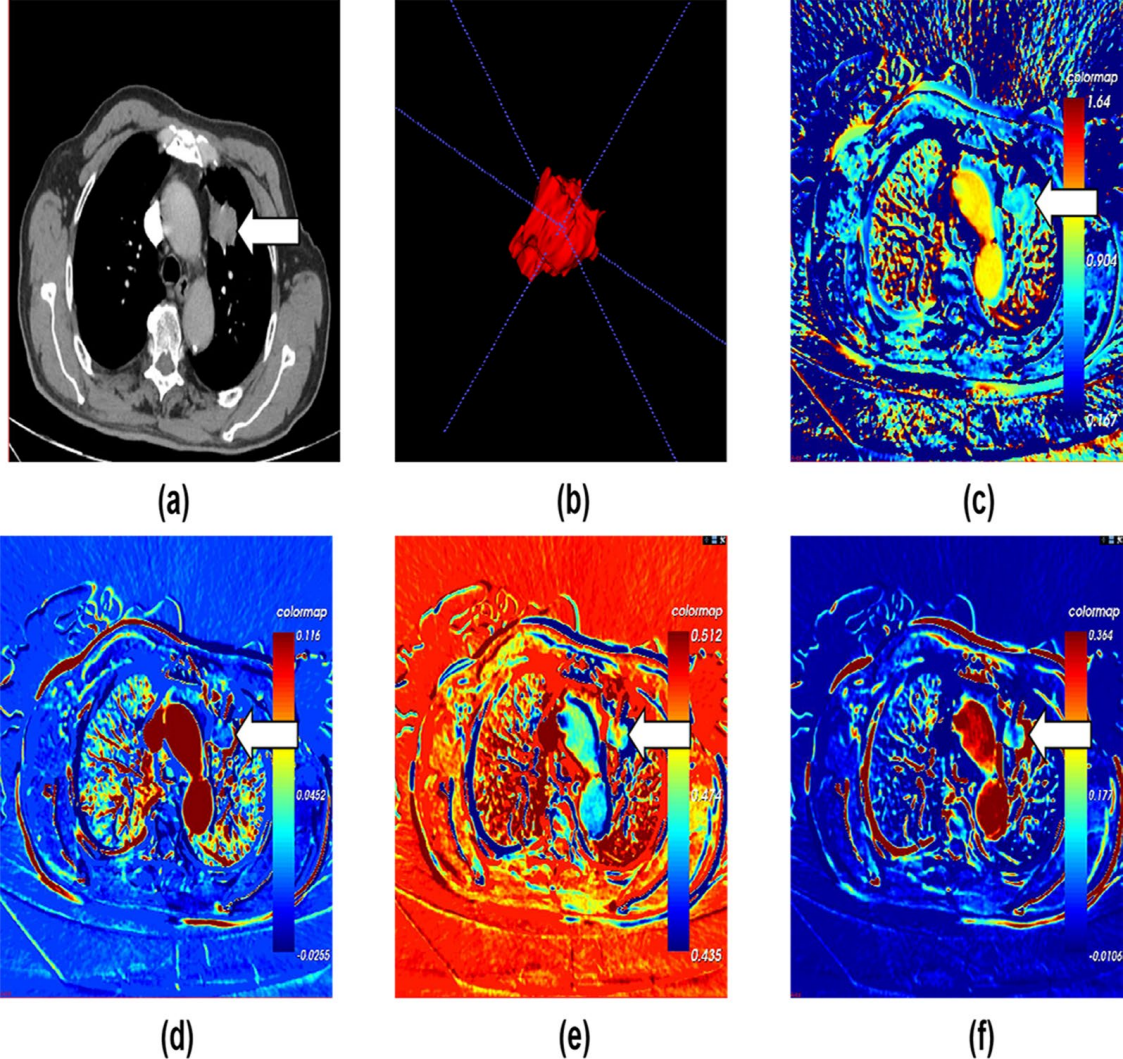
Adenocarcinoma in an 80-year-old man. **a** The mediastinal window with dynamic three stage enhanced CT scan showing a nodule in left upper lobe, the area indicated by the arrow is the tumour. **b** 3D ROI of primary tumor lesion. **c**–**f** Perfusion maps of pulmonary artery, bronchial artery, perfusion index and arterial enhancement fraction. Arrows indicate the tumours

The CT scans generated a series of images transmitted to the post-processing workstation CT Kinetics software (GE Healthcare). Identify the aorta and pulmonary stem as input arteries, and place the region of interest (ROI) in the pulmonary artery stem and lower aorta, generating a time density curves (TDC). Avoiding the visible blood vessel, calcification, liquefaction necrosis areas and normal pulmonary tissue to sketch the ROI, 3–5 layers above and below the largest tumor layer were integrated into a 3D ROI for quantitative analysis and calculation by two senior radiologists using deconvolution model method.The main parameters of the CT perfusion imaging: Median, Mean, Std, Q10, Q25, Q50, Q75, Q90 of pulmonary artery perfusion (PAP), bronchial artery perfusion (BAP), perfusion index (PI) and arterial enhancement fraction (AEF). By using the CT perfusion software, the pulmonary lesions perfusion were divided into pulmonary artery stage and aortic stage, through image reorganization and pseudocolor processing, perfusion color maps were obtained (Fig. 1). Data processing was measured three times by two senior radiologists, and the average value was taken.

### Immunohistochemical staining

The tissue in each case was fixed by 10% formalin and embedded by paraffin. Paraffin Sects (2.5 μm thickness) were used for immunohistochemistry using streptavidin peroxidase (SP) kit and a hematoxylin an eosin staining kit. The slices were deparaffinized and dehydrated in graded alcohols. Heat-induced antigen retrieval was performed by using a microwave oven and citrate buffer (pH, 6.0; 10 mol/L). All samples were immunostained by using the SP procedure with the monoclonal mouse antibodies VEGF and the monoclonal mouse antibodies CD34 (from Golden Bridge Company, Beijing). PBS (phosphate buffered saline) instead of an antibody as a negative control.

VEGF immunohistochemical scores were used immunoreactive score (IRS) for semi-quantitative determination, which synthesized the density and distribution of immunostaining. The specific calculation method is as follows: staining intensity (SI) grade: 0: no color, 1: Pale yellow, 2: Brown yellow, 3: Dark brown; Percentage of stained cells (PP): 0: no color, 1: stained cells < 10%, 2: stained cells 11–50%, 3: stained cells 51–80%, 4: stained cells > 81%, IRS = SI × PP.

Each slice was scanned at low magnification (× 10) to determined three “hot pot” areas where the number of microvessels was at maximum. In each area, one field was chosen randomly for the purpose of counting and measuring MVD. MVD were counted in the chosen field at high magnification (× 40).The field MVD value was obtained from the average MVD value in the chosen three areas.

### Statistical analysis

All data were analysed using SPSS 25.0 statistical software (SPSS for windows, version 25.0; IBM Corp, Armonk, New York). All continuous variables underwent normality test, normal distribution data were indicated with mean ± SD, abnormal distribution data were expressed as median (Q25, Q75). Mann–Whitney U test was used to test the perfusion parameters of lung benign and malignant lesions. The diagnostic efficiency of CT perfusion parameters in differential diagnosis lung lesions was assessed by receiver operating characteristic (ROC) curve. Kruskal-Wall test was used to compare the quantitative perfusion parameters with related protein expression of lung cancer with different pathological subtypes. The correlations among the CT perfusion parameters with MVD and VEGF were analysed by performing Spearman correlation analyses. *p* < 0.05 was considered statistically significant.

## Result

### Information of patients

**Fig. 2:**
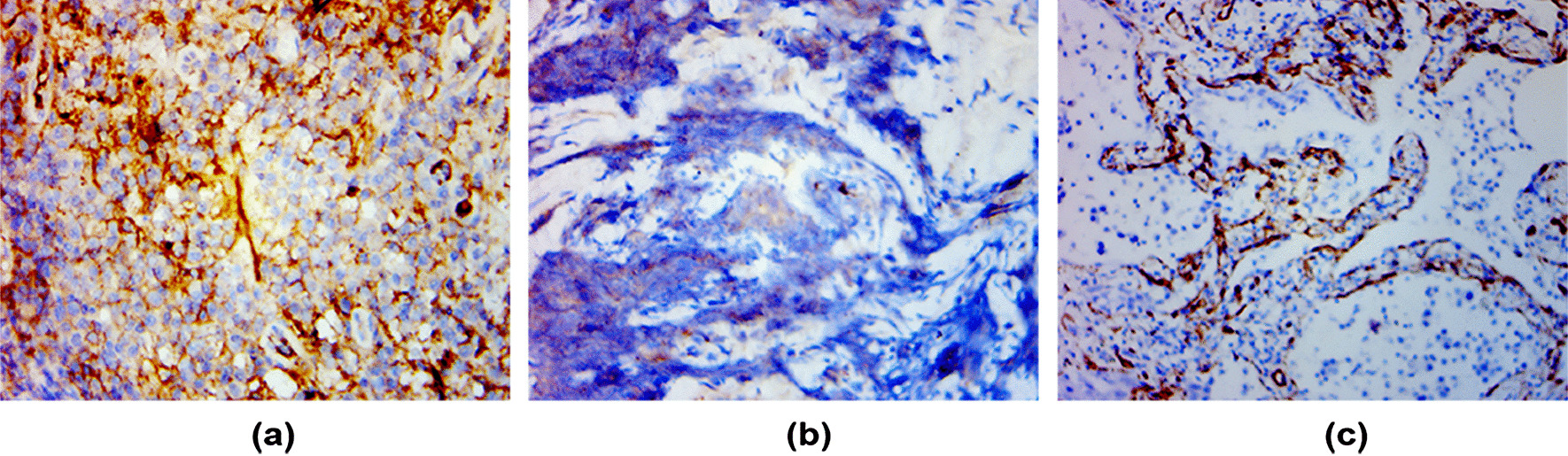
Immunohistochemical maps of lung cancer patients. **a** A squamous cell carcinoma at the lower lobe of right lung in a 67-year-old man, VEGF immunohistochemical staining of the lesion tissue, SI was 3, PP was 4, immunohistochemical score was 12. **b** A small cell carcinoma at the lower lobe of right lung in a 69-year-old man, VEGF immunohistochemical staining of the lesion tissue, SI was 2, PP was 2, immunohistochemical score was 4. **c** A adenocarcinoma in left lung in a 72-year-old woman, CD34 immunohistochemical staining image of the lesion, magnification × 400, MVD was 21

Table 1 summarizes the demographic of 73 patients with lung lesions. There were 14 cases of squamous cell carcinoma, 28 cases of adenocarcinoma and 6 cases of small cell lung cancer, 1 case of nasopharyngeal cancer metastasis, 1 large cell carcinoma, 1 spindle cell tumor, 22 case of benign (3 case of tuberculosis, 19 case of inflammatory).The median age, mean age ± standard deviation (SD) and age range was 64, (68 ± 9) and 49–85 years respectively. There was no significant difference in sex, BMI and tumor maximum diameter between malignant and benign lesions (*p* = 0.061; F = 2.045, *p* = 0.096; F = − 1.818, *p* = 0.073).Age was significant difference in lung lesions (F = 0.449, *p* = 0.001). 36 lung cancer patients with redundant sections for immunohistochemistry, 9 were squamous cell carcinoma,23 were adenocarcinoma and 4 were small cell carcinoma.(Fig. 2).

**Table 1:** Demographic of patients with lung lesions

Characteristics	Malignant (n = 51)	Benign (n = 22)	F value	*p* value
**Gender**				0.061
Male	39 (76.5%)	12 (54.5%)		
Female	12 (23.5%)	10 (45.5%)		
Age (years, x ± s)	68.2 ± 9.6	57.6 ± 11.7	0.449	< 0.001
Age range	44–85	24–75		
BMI (Kg/m2)	21.8 ± 2.4	23.0 ± 3.0	2.045	0.096
**Tumor maximum**				
Diameter (mm)	46.4 ± 19.6	37.2 ± 20.4	− 1.818	0.0.73

### CT perfusion parameters of pulmonary lesions

PAP Q10 (Z = − 2.068, *p* = 0.039), PAP Q25 (Z = − 2.044, *p* = 0.041), BAP Mean (Z = − 2.224, *p* = 0.026),BAP Std (Z = − 2.188, *p* = 0.029),BAP Q90 (Z = − 2.320, *p* = 0.020), PI Median (Z = − 2.104,* p* = 0.035),PI Mean (Z = − 2.993,* p* = 0.003), PI Q10 (Z = − 3.078, *p* = 0. 002), PI Q25 (Z = − 2.825, *p* = 0.005), PI Q50 (Z = − 2.825, *p* = 0.005) were statistically significant in differentiating benign and malignant lung lesions. PAP (Q10, Q25) of malignant lesions were higher than benign lesions, and BAP (Mean, Std, Q90) of benign lesions were higher than those of malignant lesions (see Tables 2 and 3).The parameters of PAP, BAP, PPI and AEF were not significantly different between different pathological types of lung cancer (*p* > 0.05).

**Table 2:** Perfusion parameters of PAP between benign and malignant lesions

Parameters	Benign (n = 22)	Malignant (n = 51**)**	Z value	*p* value
PAP Median	− 0.011 (− 0.069,0.050)	0.015 (− 0.002,0.042)	− 1.617	0.106
PAP Mean	− 0.024 (− 0.094,0.082)	0.028 (− 0.018,0.104)	− 1.623	0.105
PAP Std	0.096 (0.036,0.164)	0.079 (0.025,0.178)	− 0.577	0.564
PAP Q10	− 0.057 (− 0.192,0)	0 (− 0.058,0.008)	− 2.068	0.039*
PAP Q25	− 0.027 (− 0.118,0.005)	0.003 (− 0.030,0.025)	− 2.044	0.041*
PAP Q50	− 0.011 (− 0.069,0.050)	0.015 (− 0.002,0.042)	− 1.599	0.110
PAP Q75	0 (− 0.034,0.156)	0.033 (0,0.133)	− 1.431	0.153
PAP Q90	0.126 (− 0.001,0.237)	0.054 (0.001,0.360)	− 1.443	0.149

PAP,Pulmonary artery perfusion; Std,Standard deviation;* p < 0.05

**Table 3:** Perfusion parameters of PI between benign and malignant lesions

Parameters	Benign (n = 22)	Malignant (n = 51**)**	Z value	*p* value
PI Median	0.459 (0.402,0.486)	0.487 (0.466,0.498)	− 2.104	0.035*
PI Mean	0.456 (0.401,0.476)	0.483 (0.453,0.496)	− 2.993	0.003*
PI Std	0.045 (0.030,0.078)	0.019 (0.008,0.051)	− 2.921	0.003*
PI Q10	0.378 (0.298,0.444)	0.456 ()0.412,0.486	− 3.078	0.002*
PI Q25	0.429 (0.372,0.470)	0.474 (0.441,0.493)	− 2.825	0.005*
PI Q50	0.459 (0.402,0.486)	0.487 (0.466,0.498)	− 2.116	0.034*
PI Q75	0.484 (0.442,0.499)	0.494 (0.478,0.500)	− 1.467	0.142
PI Q90	0.500 (0.473,0.504)	0.500 (0.494,0.504)	− 0.168	0.866

PI,Perfusion index; Std,Standard deviation; *p < 0.05

### Diagnostic efficacy of quantitative perfusion parameters of dynamics triple-phase enhanced CT

**Fig. 3:**
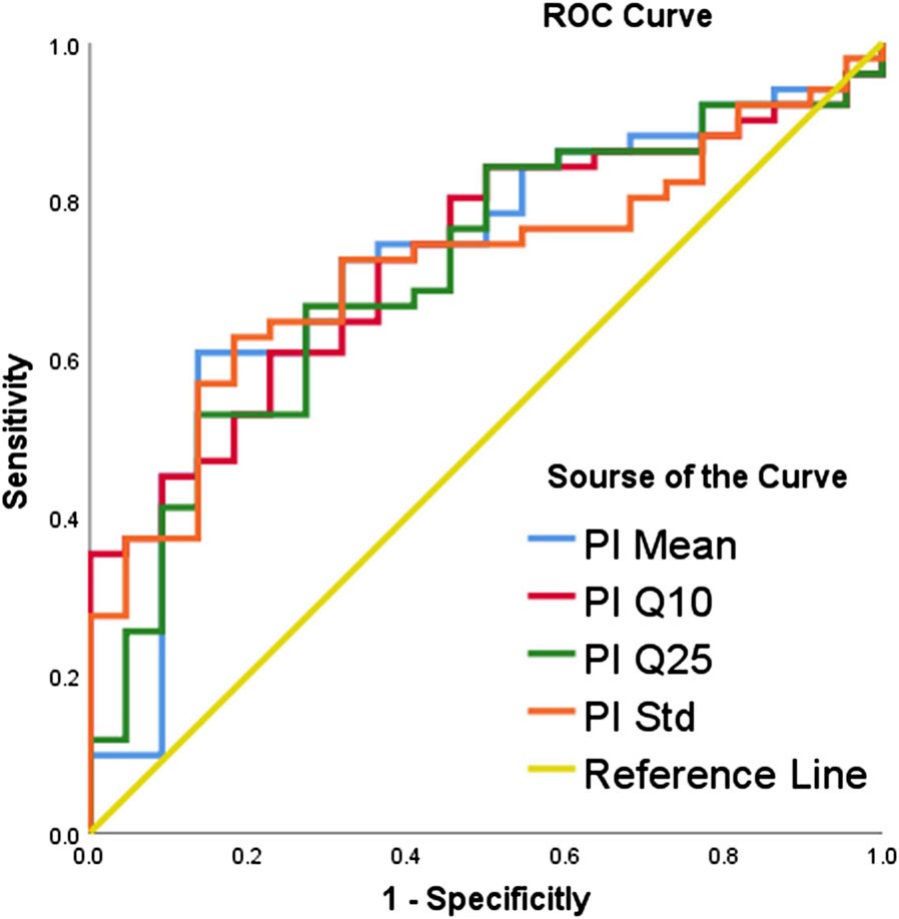
ROC curve of dynamic triple-phase enhanced CT perfusion parameters in differentiating benign and malignant lung lesions

The area under the ROC curve of PI (Std, Mean, Q10, Q25) for differentiating benign and malignant pulmonary lesions was 0.717 (95% CI = [0.598–0.835]), 0.722 (95% CI = [0.595–0.845]), 0.728 (95% CI = [0.612–0.844]), 0.709 (95% CI = [0.585–0.8 34]) respectively (Fig. 3). PI Mean, PI Q10 and PI Std have good discrimination acc -uracy. When PI Mean threshold was 0.478, sensitivity and specificity was 60.8% and 86.4% respectively; When PI Q10 threshold was 0.441, its sensitivity and specificity in differentiating malignant lesions was 60.8% and 77.3%; And when PI Std threshold was 0.029, the sensitivity and specificity was 62.7% and 81.8% respectively. When PI Q25 threshold was 0.460, sensitivity and specificity was 66.7% and 72.7% respectively.

### Correlation between CT perfusion parameters with MVD and VEGF

**Fig. 4:**
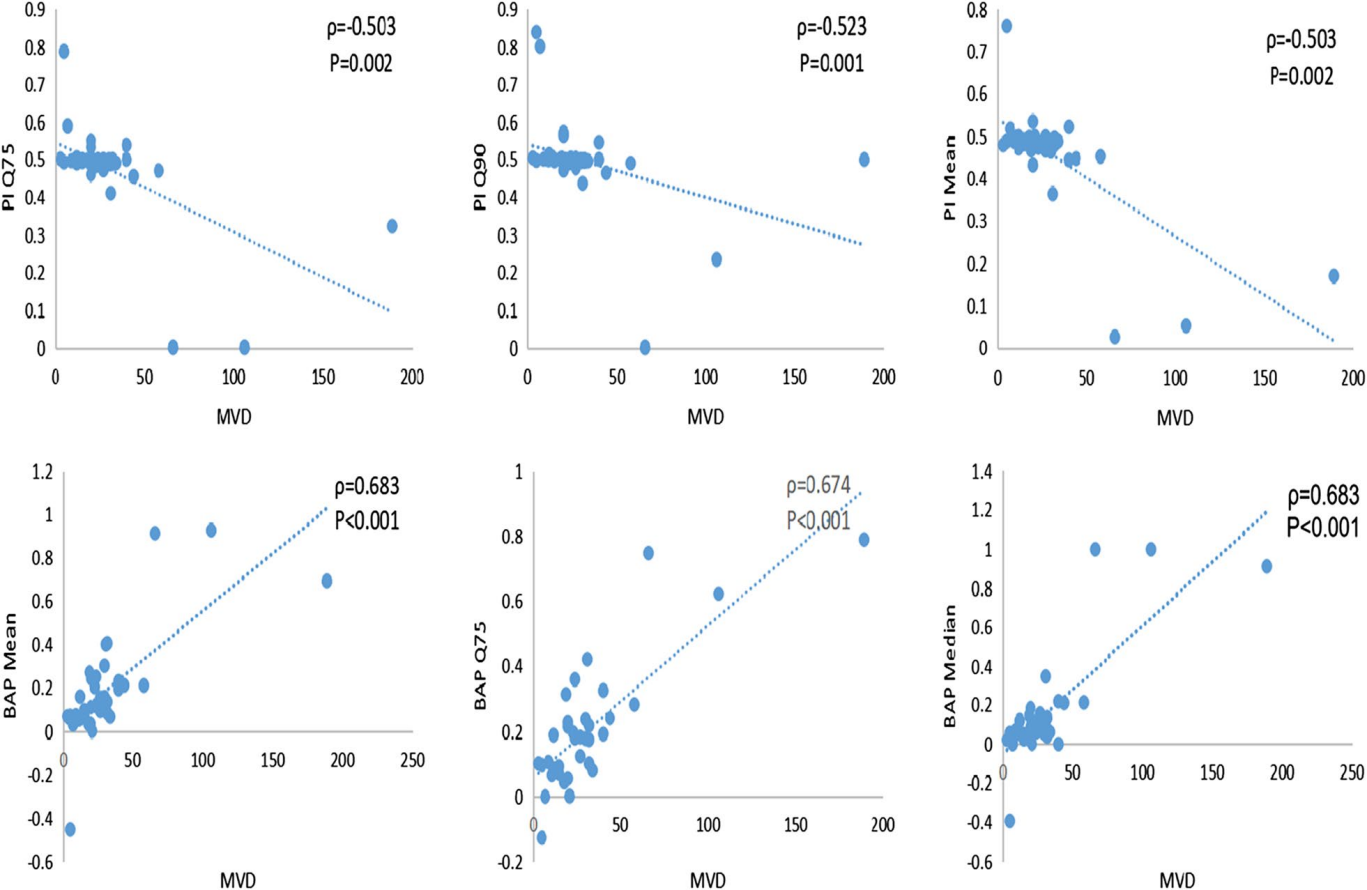
Correlation between MVD expression of lung cancer with partial perfusion parameters of dynamic triple-phase enhanced CT

**Fig. 5:**
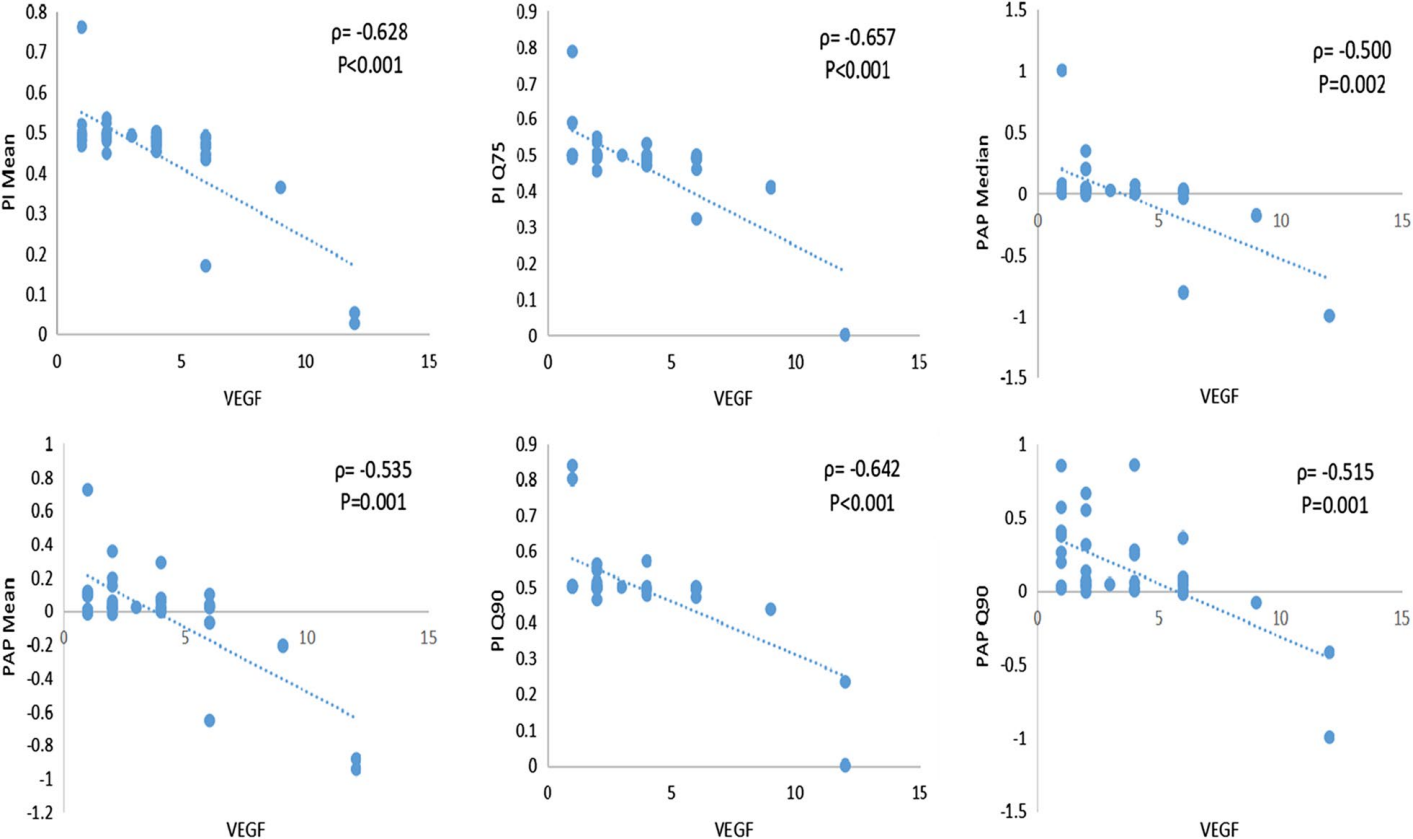
Correlation between VEGF expression of lung cancer with partial perfusion parameters of dynamic triple-phase enhanced CT

Spearman correlation coefficients (ρ) and P-values among perfusion parameters with MVD and VEGF of the lung cancer were summarized. BAP (Median, Mean, Q25, Q50, Q75, Q90) were positively correlated with MVD (ρ = 0.594, *p* < 0.001;ρ = 0.683, *p* < 0.001;ρ = 0.349, *p* = 0.037;ρ = 0.574, *p* < 0.001;ρ = 0.674, *p* < 0.001;ρ = 0.6144, *p* < 0.001). PI (Median, Mean, Q10, Q25, Q50, Q75, Q90) were negatively correlated with MVD (ρ = − 0.422, *p* = 0.010;ρ = − 0.503, *p* = 0.002;ρ = − 0.343, *p* = 0.041;ρ = − 0.486, *p* = 0.003;ρ = − 0.419, *p* = 0.011;ρ = − 0.503, *p* = 0.002;ρ = − 0.523, *p* = 0.001). AEF Q10 was positively correlated with MVD (ρ = 0.483, *p* = 0.003) (Fig. 4). The other perfusion parameters were not significantly correlated with protein expression (*p* > 0.05). PAP (Median, Mean, Q25, Q50, Q75, Q90) were negatively correlated with VEGF (ρ = − 0.500, *p* = 0.002;ρ = − 0.535, *p* = 0.001;ρ = − 0.343, *p* = 0.040;ρ = − 0.497, *p* = 0.002;ρ = − 0.493, *p* = 0.002;ρ = − 0.515, *p* = 0.001). BAP (Median, Mean, Q50, Q75, Q90) were positively correlated with VEGF (ρ = 0.573, *p* < 0.001;ρ = 491, *p* = 0.002;ρ = 0.570, *p* < 0.001;ρ = 0.488, *p* = 0.003;ρ = 0.398, *p* = 0.016). PI (Median, Mean, Q10, Q25, Q50, Q75, Q90) were negatively correlated with VEGF (ρ = − 0.602, *p* < 0.001;ρ = − 0.628, *p* < 0.001;ρ = − 0.461, *p* = 0.005;ρ = − 0.624, *p* < 0.001;ρ = − 0.607, *p* < 0.001;ρ = − 0.657, *p* < 0.001;ρ = − 0.642, *p* < 0.001) (Fig. 5). AEF Q10 was positively correlated with VEGF (ρ = 0.441, *p* = 0.007). There were no significant correlation between other parameters with the expression of VEGF (*p* > 0.05).

## Discussion

CTP is a vital method for evaluating tumor-related angiogensis, which can provide quantitative hemodynamic information of tumors. However most of the early perfusion experiments at home and abroad used 16 or 64 spiral CT to calculate the perfusion parameters, which only demonstrating the dominant circulation[20]. The study of solitary pulmonary nodules by Ohno found that DI-CTP model have better qualitative diagnostic value than the SI-CTP model[21]. Our study also found that the pulmonary artery and bronchial artery blood supply of pulmonary lesions could be observed separately based on the dual blood supply model, and parameters not only reflect the blood supply characteristics more comprehensively and accurately, but also more consistent with the growth environment and characteristics of pulmonary nodules[22, 23].

In normal conditions, the bronchial and pulmonary artery are not directly related, but when the malignant tumor continues to grow, the pulmonary artery is destroyed more and more seriously, the hyperplasia of fibers leads to the obstruction of the pulmonary artery. When the pulmonary circulation is damaged, the bronchial circulation can provide pulmonary parenchymal perfusion. Our study found that PAP (Q10, Q25),BAP (Mean, Std, Q90) and PI (Median, Mean, Std, Q10, Q25, Q50) perfusion parameters were statistically significant in the differentiation of benign and malignant lung lesions, PAP (Q10,Q25) of malignant lesions were higher than benign lesions, and BAP (Mean, Std, Q90) of benign lesions were higher than malignant lesions. In the study of Ohno[20]and Dennis[24], pulmonary artery perfusion in malignant lesions was higher than in benign lesions, which was consistent with our findings. Some studies [25–27]have found that pulmonary malignant lesions were mainly bronchial blood supply, benign lesions were mainly pulmonary artery blood supply. This was contrary to our results, maybe our study selected lung lesions larger than 2 cm in diameter, leading to an increase in pulmonary artery blood supply. Studies have shown that as the lesions grew, the proportion of pulmonary artery blood supply increased[24]. Our study suggested that the PI of malignant lesions was higher than benign lesions,the specificity of PI Mean and PI Q10 in differential diagnosis was 86.4% and 77.3%, and specificity of PI Std was 81.8% for differentiating benign lesions, which reflected the good diagnostic efficiency of identification.

Angiogenesis is a complicated process, as it is controlled by vascular growth factors and vascular growth inhibitor factors[28, 29].CTPI has received increasing clinical attention and has been used to indirectly assess tumor angiogenesis. There are many parameters to evaluate tumor angiogenesis, such as MVD, VEGF, platelet derived growth factor (PDGF) and transforming growth factor (TGF), the two most commonly used parameters MVD and VEGF are used in our research. Previous CTP imaging used a single imput model, the study between CTP parameters and MVD、VEGF of lung lesions showed that blood flow (BF) and blood volume (BV) had good correlation with MVD and VEGF[30, 31]. Our study was based on DI-CTP model by dynamic triple- phase enhanced CT to explore the correlation between its parameters with the expression of MVD and VEGF. The results showed that some scholars found that BF、PF、TPF of lung occupying lesions were positively correlated with MVD. However, the other reported that CT perfusion parameters have no relationship with MVD[32]. Our study showed that BAP related parameters were positively correlated with expression of MVD, PI related parameters were negatively correlated with MVD, among that the correlation with BAP Mean was the highest (ρ = 0.683), correlation coefficient showed that MVD had no strong correlation with CT perfusion parameters. Although the growth of malignant tumors is accompanied by a large number of neovascularization, most of these neovascularization are immaturevessels, that means, vessels with incomplete vascular structure. Our study found that there was no significant relationship between PAP and MVD in the tumor area, one reason may be the small sample size in our study; another reason may be the complicated manner in which the pulmonary artery contributes to the tumor. Li et al. found that VEGF was positively correlated with BF, BV, and MTT, and negatively correlated with TTP[33]. This study showed that the level of VEGF was positively correlated with BAP (Median、Mean、Q50、Q75、Q90) and AEF Q10, and negatively correlated with PAP (Median、Mean、Q25、Q50、Q75、Q90), PI (Median、Mean、Q10、Q25、Q50、Q75、Q90). It indicates that VEGF expressions are increased, new vessels are increased and systemic circulation is also increased.

The present study has some limitations, including the small sample size and single-center. Additionally, no further study of lung cancer with different differentiation, clinical and pathological stages. Last but not least, the increase of the equivalent dose compared with general enhanced chest CT. Therefore, further work is to expand the sample size of lung lesions with various properties and conduct a multi-center research to improve the diagnostic and differential diagnostic value of CT perfusion imaging for Pulmonary lesions.

## Conclusion

In conclusion, quantitative perfusion parameters of dynamic triple-phase enhanced CT, which can provide basis for the differential diagnosis of benign and malignant pulmonary lesions. Otherwise, dynamic triple-phase enhanced CT as a non-invasive examination method can reflect the expression of tumor cytokines related to the microvascular growth of lung cancer and the proliferation state of tumor cells, which provides a new basis and method for early diagnosis and early treatment of lung cancer patient.

## Data Availability

The datasets generated and/or analysed during the current study are not publicly available due [We will continue to expand the sample size for follow-up research] but are available from the corresponding author on reasonable request.

## Abbreviations

AEF: Arterial enhancement fraction
BAP: Bronchial artery perfusion
CTPI: CT perfusion imaging
MVD: Microvessel density
PAP: Pulmonary artery perfusion
PI: Perfusion index
ROI: Region of interest
VEGF: Vascular endothelial growth factor
